# Audit of documentation of observations on mental health services for older people (MHSOP) wards following implementation of nervecentre

**DOI:** 10.1192/bjo.2021.573

**Published:** 2021-06-18

**Authors:** Nitya Rathi

**Affiliations:** Northumbria NHS Foundation Trust

## Abstract

**Aims:**

Nervecentre is an application that can be used on mobile devices and desktop computers to record and view physical observations amongst other tools. An audit had been done previously assessing the practice of recording observations using paper documentation. That audit had recommended the use of Nervecentre to improve the recording of observations. This audit was undertaken following the introduction of Nervecentre for documentation of physical observations. The aims were to evaluate if the transition to electronic documentation of NEWS (National Early Warning Score) observations on Nervecentre has improved practice in comparison to paper documentation and to evaluate if our practice could be improved by implementing electronic observations for psychiatric observations in addition.

**Method:**

Data were collected over a 10-day period looking at all the documented observations from all inpatients on the MHSOP wards that met the inclusion criteria. Data were collected on the recording of psychiatric observations (recorded on paper charts) and physical observations (recorded on Nervecentre). The data were collated and analysed. The new data were compared to the original data from prior to the introduction of NerveCentre and the findings were presented at a local meeting.

**Result:**

This audit has highlighted that the documentation of physical observations on MHSOP wards has greatly improved since Nervecentre was introduced. There was an improvement in recording of physical observations in almost all domains measured. NEWS scores were correctly documented 100% of the time compared to 87% previously. Raised NEWS scores were correctly escalated to a senior and reviewed 80% of the time compared to 0% previously. It has also highlighted that the quality of documentation regarding psychiatric observations could be improved as we are not currently meeting local or national guidance.

**Conclusion:**

The most likely cause for the improvement in the recording of the physical observations is the implementation of Nervecentre. Nervecentre prompts users when observations are due, removes the risk of calculation errors and allows for observations to be directly escalated. Implementing Nervecentre for psychiatric observations may similarly improve the quality of these observations therefore improving patient safety.

